# Reduced immune-regulatory molecule expression on human colonic memory CD4 T cells in older adults

**DOI:** 10.1186/s12979-021-00217-0

**Published:** 2021-02-13

**Authors:** Stephanie M. Dillon, Tezha A. Thompson, Allison J. Christians, Martin D. McCarter, Cara C. Wilson

**Affiliations:** 1 Department of Medicine, University of Colorado Anschutz Medical Campus, Aurora, Colorado 80045 USA; 2 Department of Surgery, University of Colorado Anschutz Medical Campus, Aurora, Colorado 80045 USA

**Keywords:** Aging, Human, Gut, Tissue resident memory T cells, CD4 T cells, CD8 T cells

## Abstract

**Background:**

The etiology of the low-level chronic inflammatory state associated with aging is likely multifactorial, but a number of animal and human studies have implicated a functional decline of the gastrointestinal immune system as a potential driver. Gut tissue-resident memory T cells play critical roles in mediating protective immunity and in maintaining gut homeostasis, yet few studies have investigated the effect of aging on human gut T cell immunity. To determine if aging impacted CD4 T cell immunity in the human large intestine, we utilized multi-color flow cytometry to measure colonic lamina propria (LP) CD4 T cell frequencies and immune-modulatory marker expression in younger (mean ± SEM: 38 ± 1.5 yrs) and older (77 ± 1.6 yrs) adults. To determine cellular specificity, we evaluated colon LP CD8 T cell frequency and phenotype in the same donors. To probe tissue specificity, we evaluated the same panel of markers in peripheral blood (PB) CD4 T cells in a separate cohort of similarly aged persons.

**Results:**

Frequencies of colonic CD4 T cells as a fraction of total LP mononuclear cells were higher in older persons whereas absolute numbers of colonic LP CD4 T cells per gram of tissue were similar in both age groups. LP CD4 T cells from older versus younger persons exhibited reduced CTLA-4, PD-1 and Ki67 expression. Levels of Bcl-2, CD57, CD25 and percentages of activated CD38^+^HLA-DR^+^ CD4 T cells were similar in both age groups. In memory PB CD4 T cells, older age was only associated with increased CD57 expression. Significant age effects for LP CD8 T cells were only observed for CTLA-4 expression, with lower levels of expression observed on cells from older adults.

**Conclusions:**

Greater age was associated with reduced expression of the co-inhibitory receptors CTLA-4 and PD-1 on LP CD4 T cells. Colonic LP CD8 T cells from older persons also displayed reduced CTLA-4 expression. These age-associated profiles were not observed in older PB memory CD4 T cells. The decline in co-inhibitory receptor expression on colonic LP T cells may contribute to local and systemic inflammation via a reduced ability to limit ongoing T cell responses to enteric microbial challenge.

**Supplementary Information:**

The online version contains supplementary material available at 10.1186/s12979-021-00217-0.

## Background

Aging is associated with a chronic inflammatory state (“inflammaging”) which is linked to geriatric comorbidities, including cardiovascular disease, impaired mobility, cognitive decline and all-cause mortality [1, 2]. The etiology of inflammaging is likely multifactorial, but a number of animal studies have implicated a functional decline of the gastrointestinal (GI) tract as a potential driver. For example, studies in the *Drosophila* model have linked age-associated loss of intestinal barrier function to alterations in intestinal microbiota (dysbiosis), systemic metabolic defects, inflammation and age-related mortality [3, 4]. Age-associated links between enteric microbiota and local and systemic inflammation were also demonstrated in murine models [5, 6]. Older non-human primates had greater systemic inflammation, higher levels of biomarkers indicative of microbial translocation and intestinal barrier dysfunction, observations supported by increased gut permeability to large molecules [7–9]. Our previous study suggested that disruption of gut homeostasis and its link to systemic inflammation also occurs as part of human aging whereby plasma biomarkers of epithelial barrier damage and microbial translocation increased with age similar to other indicators of inflammaging (IL-6, C-reactive protein [CRP]) in persons aged 20–100 years [10]. However, few studies have investigated how aging directly impacts human intestinal immunity.

Gut T cells play critical roles in mediating both protective immunity and in maintaining gut homeostasis and epithelial barrier function (reviewed in [11]). It is therefore conceivable that alterations in the gut T cell landscape as we age could impact gut immunity against enteric pathogens as well as intestinal barrier function. Gut CD4 T cell development and their ability to induce tolerance is finely tuned by interactions between the host T cells and the local microbial community [12], yet a number of studies have associated aging with alterations in the structure of these enteric microbial communities [13] which may therefore further modulate local T cell immunity. Human gut T cells are primarily tissue-resident memory cells with distinct transcriptomic, phenotypic and functional properties compared to their blood counterparts [14–16] preventing generalization of our understanding of age effects on blood T cells to those in the gut. Indeed, the composition of naïve and memory CD4 and CD8 T cell subsets in human small and large intestine remained relatively unchanged with age; contrasting with decreases in naïve T cells and increases in effector memory subsets in peripheral blood (PB) and other lymphoid and mucosal sites [16, 17].

In a recent study investigating the impact of age on human small intestine T cells, we demonstrated that jejunum lamina propria (LP) CD4 T cells from older persons (≥65 yrs) displayed phenotypic and functional differences versus those from younger persons (≤45 yrs) including reduced expression of the co-inhibitory molecule CTLA-4, increased spontaneous cell death and reduced frequencies of T helper 17 cells [18]. Utilizing in situ imaging, Senda et al. found an age-dependent decrease in naïve CD4 and CD8 T cell frequencies located in isolated lymphoid follicles (ILFs) within jejunum tissue, but frequencies in colonic ILFs were unchanged [19] signifying that age effects may differ throughout the intestinal tract. In fact, the GI tract is known for its regional specialization with distinctive differences in anatomical structure, distribution of innate and adaptive immune cells and in the local composition of the various microbial species [20, 21]. Therefore, to expand on our previous work investigating small intestinal T cells, we undertook this exploratory study to evaluate the impact of age on the frequency and immune phenotype of human colon LP CD4 T cells obtained from younger persons aged ≤45 yrs and older persons aged ≥65 yrs. To determine whether aging-related findings were gut T cell subset-specific, we also evaluated the frequency and phenotypic profiles of human LP CD8 T cells. Finally, given that gut T cells are primarily tissue-resident memory cells, we also probed the potential tissue specificity of the age-associated phenotype by investigating the same profiles in memory PB CD4 T cells in a separate cohort of similarly aged younger and older persons.

## Results

### Colonic LP CD4 T cells from older persons exhibit reduced CTLA-4, PD-1 and Ki67 expression

**Fig. 1:**
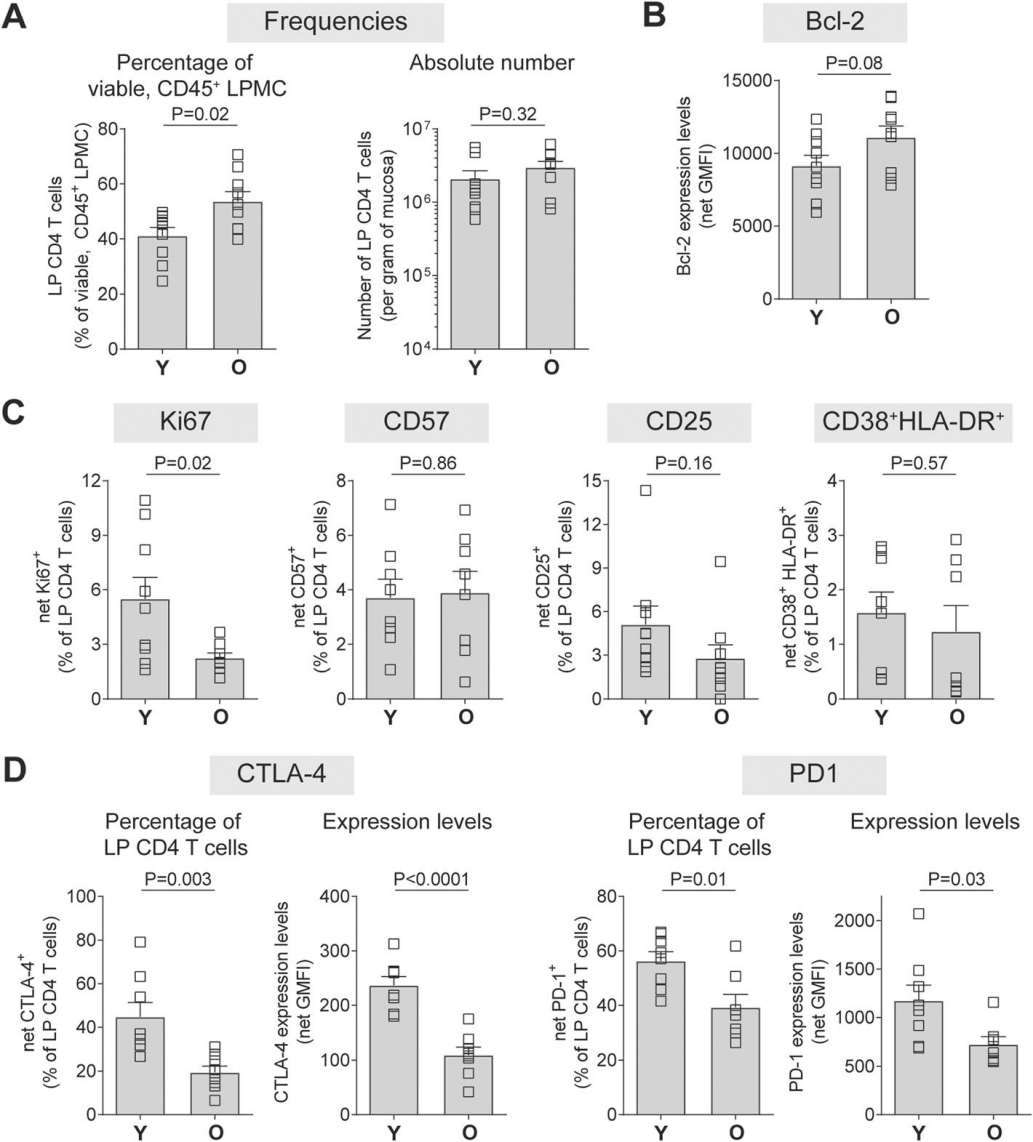
Age is associated with alterations in phenotypic profiles of human LP CD4 T cells. LPMC were isolated from colonic tissue samples obtained from younger (Y; ≤45 yrs) and older (O; ≥65 yrs) persons (*N* = 9/age group unless otherwise stated) and multi-color flow cytometry used to evaluate **a** baseline frequencies and baseline expression of **b** Bcl-2, **c** CD38 and HLA-DR (Y *N* = 8; O *N* = 7), CD25, Ki67 (O N = 8), CD57 (Y N = 8; O N = 7) or **d** CTLA-4 (Y N = 8; O N = 8) and PD-1 (Y N = 8; O N = 7). Frequency values are shown as a percentage of LP CD4 T cells in viable, CD45^+^ LPMC or as absolute number per gram and phenotypic expression as the percentage of LP CD4 T cells expressing each marker or as total expression levels on LP CD4 T cells (Geometric Mean Fluorescence Intensity; GMFI). Isotype control (Bcl-2, CD38, Ki67, CTLA-4), Fluorescence minus one (FMO; CD25) or FM4 (HLA-DR, CD57, PD-1) values have been removed (net). Bar graphs represent mean ± SEM with individual samples shown as open squares. Statistical analysis: unpaired t-tests

Multicolor flow cytometry was used to evaluate baseline frequencies and immune phenotypic profiles of LP CD4 T cells from younger (≤45 yrs) and older donors (≥65 yrs) (Additional file 1: Figure S1). Frequencies of total LP CD4 T cells, as a fraction of total viable, CD45^+^ LPMC, were significantly higher in older samples compared with younger samples (Fig. 1a). However, the absolute number of LP CD4 T cells normalized to gram of tissue, was similar between younger and older samples (Fig. 1a).

To determine the effect of age on LP CD4 T cell phenotype, canonical indicators of T cell survival (anti-apoptotic/pro-survival Bcl-2), proliferation (Ki67), senescence (CD57), activation (CD25 [IL-2Rα]; CD38 and HLA-DR co-expression) and negative regulation (CTLA-4, PD-1, Tim-3, Lag-3) were measured ex vivo (baseline) and compared between younger and older samples. On average, most LP CD4 T cells from both younger (mean ± SEM: 99.72 ± 0.07%) and older (99.68 ± 0.07%) samples expressed Bcl-2 (Additional File 1: Fig. S1b). Levels of Bcl-2 expression, measured as geometric mean fluorescence intensity (GMFI), were higher in older (11,108 ± 779 GMFI) compared to younger samples (9147 ± 727 GMFI), but this did not reach statistical significance (*P* = 0.08) (Fig. 1b). In younger samples, Ki67 was expressed by 5.5% ± 1.2% of total LP CD4 T cells and significantly fewer LP CD4 T cells expressed this proliferation marker in older samples (2.2 ± 0.3%) (Fig. 1c). Percentages of LP CD4 T cells expressing CD57, CD25 or co-expressing CD38 and HLA-DR (CD38^+^ HLA-DR^+^) were not statistically different between younger and older samples (Fig. 1c).

The percent of LP CD4 T cells expressing CTLA-4 was significantly lower in older samples (19.4 ± 2.9%) compared to younger samples (44.8 ± 6.6%; *P* = 0.003) (Fig. 1d). Overall expression levels of CTLA-4 were also significantly lower in older samples (O: 110 ± 14 GMFI; Y: 237 ± 16 GMFI; *P* < 0.0001) (Fig. 1d). Both the percentage of older LP CD4 T cells expressing PD-1 (39.3 ± 4.8%) and overall expression levels (722 ± 82 GMFI) were significantly lower than on younger LP CD4 T cells (56.3 ± 3.4%, *P* = 0.01; 1173 ± 161 GMFI, *P* = 0.03) (Fig. 1d). On average, < 0.5% of LP CD4 T cells expressed Tim-3 in both younger (0.4 ± 0.1%) and older (0.3 ± 0.1%) samples with no age-effect observed (*P* = 0.65) (data not shown). Similarly, Lag-3 was expressed by few LP CD4 T cells in younger (0.8 ± 0.2%) and older (0.5 ± 0.2%) samples with no statistical difference observed (*P* = 0.22) (data not shown).

### PB memory CD4 T cells from older persons display increased CD57 expression

**Fig. 2:**
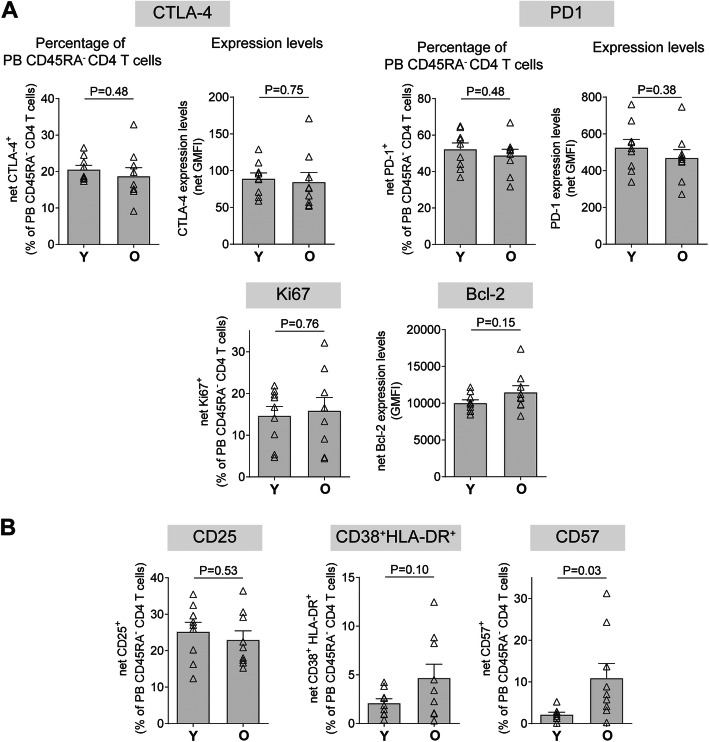
PB CD45RA- CD4 T cells have limited age-associated alterations in immune phenotype. PBMC were isolated from blood samples obtained from younger (Y; ≤45 yrs) and older (O; ≥65 yrs) persons (*N* = 9/age group unless otherwise stated) and multi-color flow cytometry used to evaluate baseline expression of a CTLA-4, PD-1, Ki67, Bcl-2 or b CD25, CD38 and HLA-DR, or CD57 (Y *N* = 8). Values are shown as the percentage of LP CD4 T cells expressing each marker or as total expression levels on LP CD4 T cells (Geometric Mean Fluorescence Intensity; GMFI). Isotype control (Bcl-2, CD38, Ki67, CTLA-4), Fluorescence minus one (FMO; CD25) or FM4 (HLA-DR, CD57, PD-1) values have been removed (net). Bar graphs represent mean ± SEM with individual samples shown as open triangles. Statistical analysis: unpaired t-tests

We next evaluated expression of the same panel of makers in PB CD4 T cells from a separate cohort of younger (≤45 yrs) and older (≥65 yrs) donors. Given that the majority of LP CD4 T cells (89.1 ± 4.2%) expressed markers indicative of an effector memory cell phenotype (CD45RA^−^CD62L^−^) (Additional File 2: Figure S2a), the age effects on phenotypic marker expression of PB CD4 T cells were evaluated in PB CD4 T cells enriched for memory cells based on lack of CD45RA expression (Additional File 2: Figure S2b). Frequencies of PB CD45RA^−^ CD4 T cells were not significantly different between younger and older samples evaluated either as the fraction of total memory cells within total CD4 T cells or within total viable PB mononuclear cells (PBMC) (Additional File 2: Figure S2c). In contrast to older LP CD4 T cells, expression of CTLA-4 and PD-1 were similar between younger and older PB CD45RA^−^ CD4 T cells (Fig. 2a). Age effects were also not observed for PB CD45RA^−^ cells expressing Ki67 or Bcl-2. Similar to LP CD4 T cells, percentages of CD25^+^ or CD38^+^HLA-DR^+^ PB CD45RA^−^ CD4 T cells were not significantly different between the age groups. In contrast to LP CD4 T cells, a greater fraction of PB CD45RA^−^ CD4 T cells expressed CD57 in older versus younger samples (Fig. 2b).

### Colonic CD8 T cells have distinct immune phenotypes compared to colonic CD4 T cells

To determine if age-effects noted in colon LP CD4 T cells were also reflected in colon CD8 T cells, we next evaluated frequency and phenotype of total CD8 T cells in the same colon samples in which we had analyzed CD4 T cell profiles (Additional File 1: Figure S1a; Additional File 3: Figure S3a). Of note, the majority of LP CD8 T cells in younger persons were also effector memory T cells (65.9 ± 7.9%) (Additional File 3: Figure S3b). However, this fraction of memory cells in CD8 T cells was significantly lower than the percentage of effector memory LP CD4 T cells (Additional File 3: Figure S3c). A greater fraction of CD8 T cells were terminally differentiated effector memory (TDEM; CD45RA^+^CD62L^−^) cells versus LP CD4 T cells. Further comparisons between younger colon CD8 and CD4 T cells highlighted additional differences between these two cell populations despite both residing at the same tissue site (Additional File 4: Table S1). Frequencies of CD8 T cells were significantly lower than CD4 T cells and, on average, only constituted 14% of total viable CD45^+^ LPMC versus 41% for CD4 T cells and 22.3% of total LP CD3^+^ T cells versus 70.1% for CD4 T cells. CD8 T cells had lower expression of Bcl-2 than CD4 T cells and fewer CD8 T cells expressed CD25, CTLA-4 and PD-1. Conversely, a greater percentage of LP CD8 T cells were CD38^+^HLA-DR^+^ and expressed CD57.

### Colonic CD8 T cells from older persons have lower levels of CTLA-4 but not PD-1

**Fig. 3:**
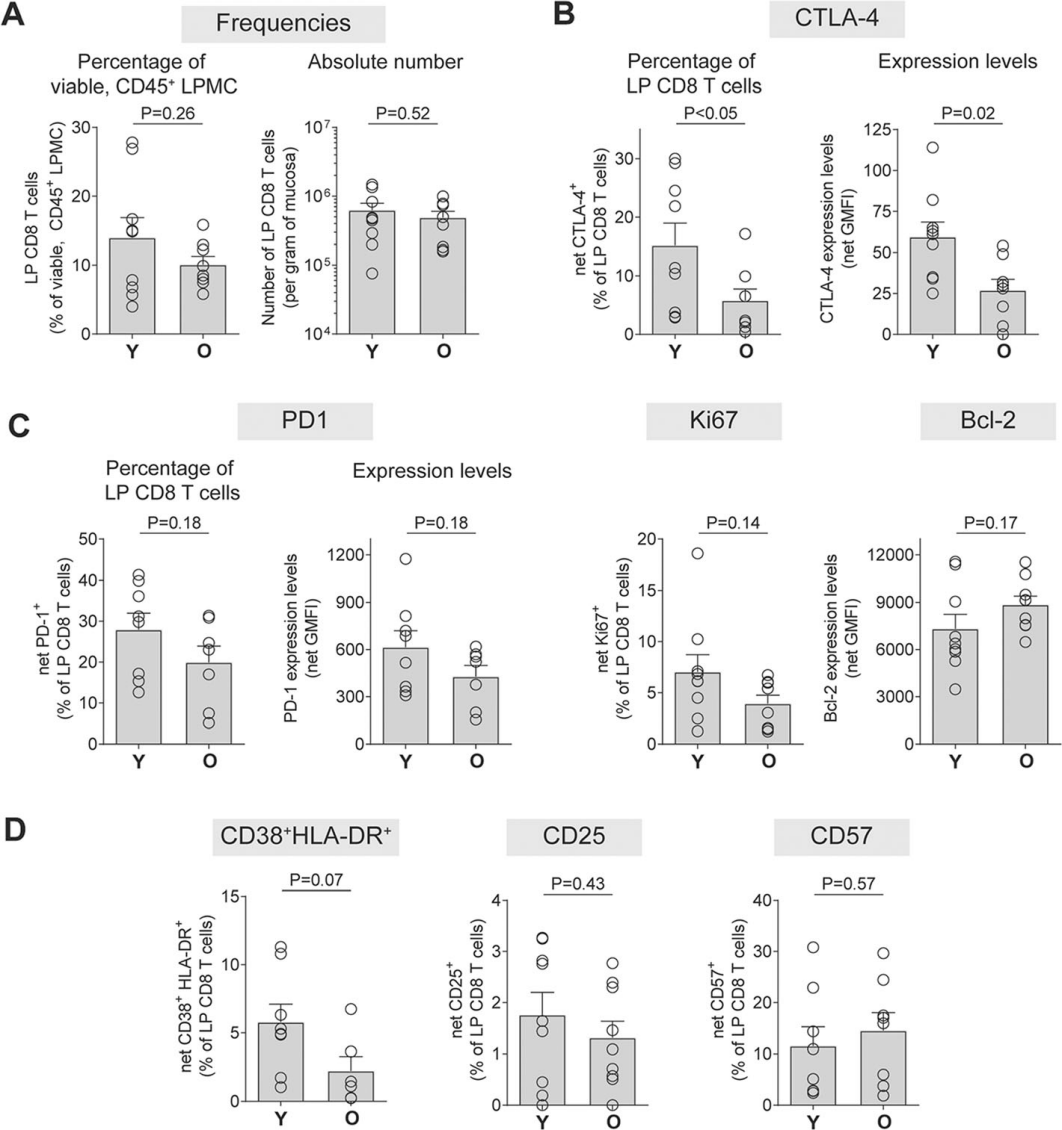
Age effects on LP CD8 T cells. LPMC were isolated from colonic tissue samples obtained from younger (Y; ≤45 yrs) and older (O; ≥65 yrs) persons (*N* = 9/age group unless otherwise stated) and multi-color flow cytometry used to evaluate **a** baseline frequencies and baseline expression of **b** CTLA-4 (O N = 8), **c** PD-1 (Y N = 8; O N = 8), Ki67 (O N = 8), Bcl-2, and **d** CD38 and HLA-DR (Y N = 8; O *N* = 6), CD25, and CD57 (Y N = 8; O N = 8). Frequency values are shown as a percentage of LP CD4 T cells in viable, CD45^+^ LPMC or as absolute number per gram and phenotypic expression as the percentage of LP CD8 T cells expressing each marker or as total expression levels on LP CD8 T cells (Geometric Mean Fluorescence Intensity; GMFI). Isotype control (Bcl-2, CD38, Ki67, CTLA-4), Fluorescence minus one (FMO; CD25) or FM4 (HLA-DR, CD57, PD-1) values have been removed (net). Bar graphs represent mean ± SEM with individual samples shown as open circles. Statistical analysis: paired t-tests

Frequencies of LP CD8 T cells, either as total number of cells per gram of tissue or fraction of viable LPMC, were not significantly different between younger and older samples (Fig. 3a). Percentages and expression levels of CTLA-4 were significantly lower in LP CD8 T cells from older samples (Fig. 3b), similar to the age effect observed in LP CD4 T cells (Fig. 1). No statistically significant differences in PD-1, Ki67 or Bcl-2 expression by LP CD8 T cells in younger and older samples were noted (Fig. 3c). Although the percentage of LP CD8 T cells co-expressing CD38 and HLA-DR was, on average, lower in samples from older persons (2.2 ± 1.0%) versus younger samples (5.8 ± 1.3%), this difference did not reach statistical significance (*P* = 0.07) (Fig. 3d). Similar to LP CD4 T cells, expression of CD25 and CD57 was not statistically different between younger and older samples.

## Discussion

It is now well accepted that peripheral blood CD4 and CD8 T cells undergo dynamic changes as humans age, and these changes likely have critical consequences on the ability to mount immune responses against pathogens as well as develop effective vaccine responses (reviewed in [22–24]). However, recent studies have underlined the inherent differences between circulating and tissue-resident T cells, including those that reside in the gut mucosa [14, 25], highlighting that care must be taken in simply extrapolating what we know about age effects on blood T cells to those in other tissues. To further our understanding of the potential impact of aging on human colon CD4 T cells, we undertook this exploratory study to measure expression levels of a panel of molecules generally considered as canonical indicators of blood T cell survival, proliferation, senescence, activation and negative regulation directly ex vivo in colon tissue samples obtained from younger and older persons.

Our study demonstrated that in older persons, LP CD4 T cells displayed features of a dysregulated phenotype primarily characterized by decreased expression of CTLA-4 and PD-1, molecules typically associated with limiting CD4 T cell activation. We have previously observed lower expression of CTLA-4 on LP CD4 T cells obtained from jejunum samples of a different cohort of older persons [18], suggesting an age effect may occur, at least in the context of CTLA-4 expression, throughout the GI tract. Notably, in this current study, we did not observe differences in expression of CTLA-4 or PD-1 between younger and older memory PB CD4 T cells (albeit from unmatched donor blood samples), potentially highlighting a gut-specific effect. Although previous studies have noted an age-associated increase in CTLA-4 or PD-1 expression on blood CD4 T cells [26–29], the lack of an apparent age effect observed here is in agreement with a number of other studies [30–33] and may relate to study populations (e.g. age range, sex) and/or the type of CD4 T cell evaluated (e.g. total versus memory populations).

CTLA-4 and PD-1 are inhibitory receptors that serve as regulators of T cell activation. In healthy persons (aged 57-65 yrs), higher percentages of CD4 T cells expressing CTLA-4 were noted in ileum and rectal biopsies versus CD4 T cells isolated from PB [34]. We previously postulated that high expression of these immune-inhibitory receptors in the gut may be an important regulatory mechanism to limit unnecessary CD4 T cell activation in response to the local enteric commensal microbial community [18]. Certainly, their critical role in controlling gut T cell immunity is highlighted by recent observations that cancer-based immunotherapies designed to block these molecules can sometimes lead to unintended immune-mediated gut inflammation [35, 36]. A number of studies have linked age-associated systemic inflammation to gut epithelial barrier dysfunction, local inflammation and/or dysbiosis [3–10]. Therefore, it is tempting to speculate that an inability to limit inflammatory colonic CD4 T cell responses to translocating bacteria due to decreased signaling through CTLA-4 and PD-1 might contribute to an inflammatory gut environment in older persons. However, future studies are needed to determine the functional outcomes of reduced expression of these molecules on colonic LP CD4 T cells as we age. Although both molecules are generally grouped together as “inhibitory receptors”, how this regulation of T cell function is achieved differs between the two, both with timing of expression and the ligands that activate their respective signaling pathways [37, 38]. Thus, the downstream consequences of lower expression of each of these molecules will likely be differentially influenced by age-associated intrinsic and extrinsic factors.

It will also be important to determine what mechanisms drive these altered profiles associated with aging. Human gut T cells are primarily tissue-resident memory cells, suggesting that these driving factors would likely be a part of the local environment [14–16]. However, gut-associated inflammation has been linked to the homing of PB CD4 T cells into gut tissue, particularly to gut-associated lymphoid tissue [39, 40]. This raises the interesting possibility that the aging-associated profiles may primarily be expressed on recently recruited PB CD4 T cells. Similar to the functional consequences of reduced regulatory molecule expression, the mechanisms driving reduced regulatory molecule expression will likely be multifactorial.

In addition to reduced expression of co-inhibitory receptors, significantly fewer LP CD4 T cells in older persons expressed Ki67, an indicator of homeostatic proliferation and cell turnover. However, the proportion of Ki67-expressing LP CD4 T cells was generally low in younger donors (average < 5.5%). Although this finding suggests that LP CD4 T cells normally exist in a more quiescent state with limited cellular turnover compared to circulating memory CD4 T cells, it is difficult to determine whether the further reduction observed in older samples has biological significance.

A number of recent studies have highlighted that the function of gut CD8 T cells, and their ability to respond to pathogenic and tumor challenge, is also influenced by the enteric commensal microbial community [41, 42]. These studies prompted us to investigate the impact of age on human colonic CD8 T cells. Quantitative and qualitative differences between CD8 and CD4 T cells from donor-matched younger samples were noted. CD8 T cells constituted a much smaller fraction of total T cells in colonic LPMC, and a greater fraction expressed canonical makers of activation (CD38^+^HLA-DR^+^) and senescence (CD57). Conversely, levels of the co-inhibitory molecules CTLA-4 and PD-1 were significantly lower on CD8 T cells relative to their CD4 counterparts. These distinct phenotypes may reflect functional differences between the two T cell populations with gut resident CD8 T cells primarily being involved in host defense while LP CD4 T cells also have additional roles in homeostasis and may require greater regulation. However, despite the differential expression patterns between the two T cell populations, the age effects were generally similar with lower expression of CTLA-4, PD-1 and homeostatic turnover (Ki67) although the latter two age effects were more pronounced for older CD4 T cells. Thus, the factors driving age-associated gut T cell dysregulation may be similar for both CD4 and CD8 T cells and both populations may contribute to an inflammatory state in older persons.

This study is exploratory in nature due to having a relatively small sample size in each age group, to having patient mismatched gut and blood samples, and to the lack of detailed clinical information, including other co-morbidities and medication use, for the study cohorts. A larger, well-controlled clinical study that includes participant-matched colonic tissue and blood samples and comprehensive clinical information is required to fully confirm our tissue-specific and age-related findings. Furthermore, given the recent observation of an age-associated decline of PD-1-expressing human PB CD4 T cells in specific memory subsets of older females [29], it will be important to determine if age and sex intersect to differentially impact phenotype and function of LP CD4 and CD8 T cells.

## Conclusions

Although the effects of aging in humans are well established for circulating CD4 and CD8 T cells, the inherent differences between blood and tissue-resident immune cells prevents us from extrapolating this to gut tissue-resident memory T cell populations. We had previously shown that small intestinal CD4 T cells from older persons displayed a dysregulated phenotype with decreased expression of the co-inhibitory molecule CTLA-4. We now expand on these findings and demonstrate that large intestinal CD4 and CD8 T cells also display an altered immune phenotype consistent with immune dysregulation with aging. In the GI tract, T cells are critical for both immunity and maintaining homeostasis, so alterations with age may have significant impacts on gut health. Our study provides the ground work for future investigations into the impact of age on gut T cell immunity to definitively link changes in T cell phenotype and function to local inflammation and epithelial barrier breakdown that lead to increased systemic inflammation and age-associated co-morbidities.

## Methods

### Collection and isolation of colon LP mononuclear cells (LPMC)

Human colon tissue samples were obtained through the University of Colorado Anschutz Medical Campus from patients scheduled for elective abdominal surgery. Tissue samples were obtained from surgical anastomotic junctions and were macroscopically healthy and normal in appearance. No samples were obtained from patients with a history of Inflammatory Bowel Disease, HIV-1 infection, treatment with immunosuppressive drugs, or recent chemotherapy (within 8 weeks). All patients voluntarily gave informed consent to permit unrestricted use of the samples for research purposes. Protected patient information for all samples was de-identified to the investigators and only age, sex and reason for surgery were provided (Additional File 5: Table S2) with the majority of samples in both younger and older groups obtained from persons undergoing elective surgery for gastrointestinal-associated cancers. Research associated with the use of LPMC was reviewed by the Colorado Multiple Institutional Review Board (COMIRB) at the University of Colorado Anschutz Medical Campus and deemed Not Human Subject Research as defined by their polices in accordance with OHRP and FDA regulations. For studies investigating frequencies and phenotypic profiles of CD4 and CD8 T cells, a total of 18 colonic samples were obtained with 9 samples obtained from younger persons (mean age: 37 yrs., range 27–44; 5 males, 4 females) and 9 samples obtained from older persons (mean age: 80 yrs., range 72–88; 6 males and 3 females). For measurement of memory CD4 and CD8 T cell populations, samples were obtained from younger donors only (*N* = 6; mean 40 yrs., range 32–44; 2 males, 4 females). LPMCs were isolated from the tissue samples in a two-step procedure with removal of the epithelial layer and then disassociation of the LP layer into single cells using Collagenase D (Sigma-Aldrich, St Louis, MO) prior to cryopreservation, as previously described [18, 43–47].

### Collection and isolation of PBMC

Human PBMC samples were selected from an ‘in house” biorepository of cryopreserved samples obtained from donors identifying as healthy and HIV-1 negative. Donors were recruited from the local research community and through the University of Colorado Hospital (UCH) Internal Medicine Clinic; due to the retrospective nature of this study, these donors were recruited under a consent process that did not include access to medical records and only age and sex were recorded [10]. All the study subjects participated voluntarily and gave written, informed consent for use of the isolated PBMC for research studies. This study was approved by COMIRB. A total of 18 PBMC samples were obtained with 9 samples obtained from younger persons (mean age: 33 yrs., range 27–40; 4 males, 5 females) and 9 samples obtained from older persons (mean age: 79 yrs., range 68–91; 6 males and 3 females). PBMC were isolated using standard Ficoll-Hypaque density gradient centrifugation, cryopreserved and stored in liquid nitrogen as previously detailed [48–50].

### Surface and intracellular multi-color flow cytometry staining

All antibodies and dyes used in the various multi-color flow cytometry staining panels are detailed in Additional File 6: Table S3. LPMCs and PBMCs were stained with viability dye and various combinations of antibodies directed against surface molecules (CD45, CD3, CD4, CD8, CD45RA, CD62L, CD57, CD38, HLA-DR, CD25, PD-1, LAG-3, TIM-3) followed by intracellularly staining for Bcl-2, Ki67 or CTLA-4, using Foxp3/Transcription Factor Staining Buffers (eBioscience, Invitrogen) as previously detailed [18]. Multiple staining panels were used to accommodate the listed fluorochromes. In some instances, low cell yields prevented staining with all panels.

### Flow cytometry acquisition and analysis

All flow cytometry data were collected using the LSRII flow cytometer (BD Biosciences, San Jose, CA) and the BD FACSDiva software (v9, BD Biosciences). Quality control measures were completed daily as detailed [18]. FlowJo™ Software for Windows (v10.6.2, Ashland, OR) was used to analyze all flow cytometry data. Flow-cytometry gating strategies for LP T cells are detailed in Additional File 1: Figure S1A. LP CD3^+^ CD4^+^ and CD3^+^ CD8^+^ T cells were identified within CD45^+^, viable, single lymphocytes (based on forward and side scatter properties). Control and antibody expression profiles are shown for Bcl-2, Ki67, CD57, CD25, CD38, HLA-DR, CTLA-4 and PD-1 by CD4 T cells in Additional File 1: Figure S1B, by CD8 T cells in Additional File 4: Figure S3A) and for identification of memory T cell subsets as effector memory (CD45RA^−^ CD62L^−^), central memory (CD45RA^−^ CD62L^+^), terminally-differentiated effector memory (CD45RA^+^and CD62L^−^), and naïve (CD45RA^+^ CD62L^+^) for CD4 (Additional File 2, Figure S2A) and CD8 (Additional File 4: Figure S3B) T cells. PB CD4 T cells were identified within an initial lymphocyte gate based on forward-scatter and side-scatter properties and then as viable, single CD3^+^ lymphocytes (Additional File 2: Figure S2B). Memory PB CD4 T cells were identified as CD45RA^−^. For phenotypic analysis of LP CD4 and CD8 T cells, gates were established on isotype control staining for CD45RA, CD62L, Bcl-2, Ki67, CD38, Lag-3 and CTLA-4, Fluorescence minus one (FMO) for CD25 or FM4 for CD57, HLA-DR and PD-1. For PB CD4 T cells, isotype controls were used to determine specific staining for CD45RA, Bcl-2, Ki67, CD38, HLA-DR, CTLA-4, PD-1, Lag-3, Tim-3 and FMO for CD25 and CD57.

### Statistical analyses and graphing

Statistical analyses and graphing were performed on GraphPad Prism for Windows (v8.4.1, GraphPad Software, La Jolla, CA). Unpaired t-tests were performed to determine statistical differences between the age groups. Paired t-tests were performed to determine differences between donor-matched LP CD4 and CD8 T cells. Sample sizes (N), means and standard error of the mean (SEM) are displayed in the figure legends. Outliers were identified using the Rout Method and removed from the analyses.

## Supplementary Information

**Additional file 1: Figure S1.** Multi-color flow cytometry profiles to enumerate frequencies and phenotypic profiles of human colon LP CD4 T cells.

**Additional file 2: Figure S2.** LP and PB memory CD4 T cell profiles.

**Additional file 3: Figure S3.** Multi-color flow cytometry profiles to enumerate frequencies and phenotypic profiles of human colon LP CD8 T cells.

**Additional file 4: Table S1.** Frequencies and expression of survival, activation and immune-regulatory markers by LP CD8 T cells or LP CD4 T cells in younger persons.

**Additional file 5: Table S2.** Patient details for procured colonic tissue samples.

**Additional file 6: Table S3.** Antibodies and dyes used for multi-color flow cytometry.

## Data Availability

The datasets used and/or analyzed during the current study are available from the corresponding author on reasonable request.

## Abbreviations

LP: Lamina propria
LPMC: Lamina propria mononuclear cells
PB: Peripheral blood
PBMC: Peripheral blood mononuclear cells
GMFI: Geometric mean fluorescence intensity
GI: Gastrointestinal
EM: Effector memory
CM: Central memory
TDEM: Terminally differentiated effector memory
