# Supplementary figures and images for: Reduced immune-regulatory molecule expression on human colonic memory CD4 T cells in older adults
Source: Immun Ageing. 2021 Feb 13;18:6. doi: 10.1186/s12979-021-00217-0 (PMC7881462; doi:10.1186/s12979-021-00217-0)

# **Additional File 1.**

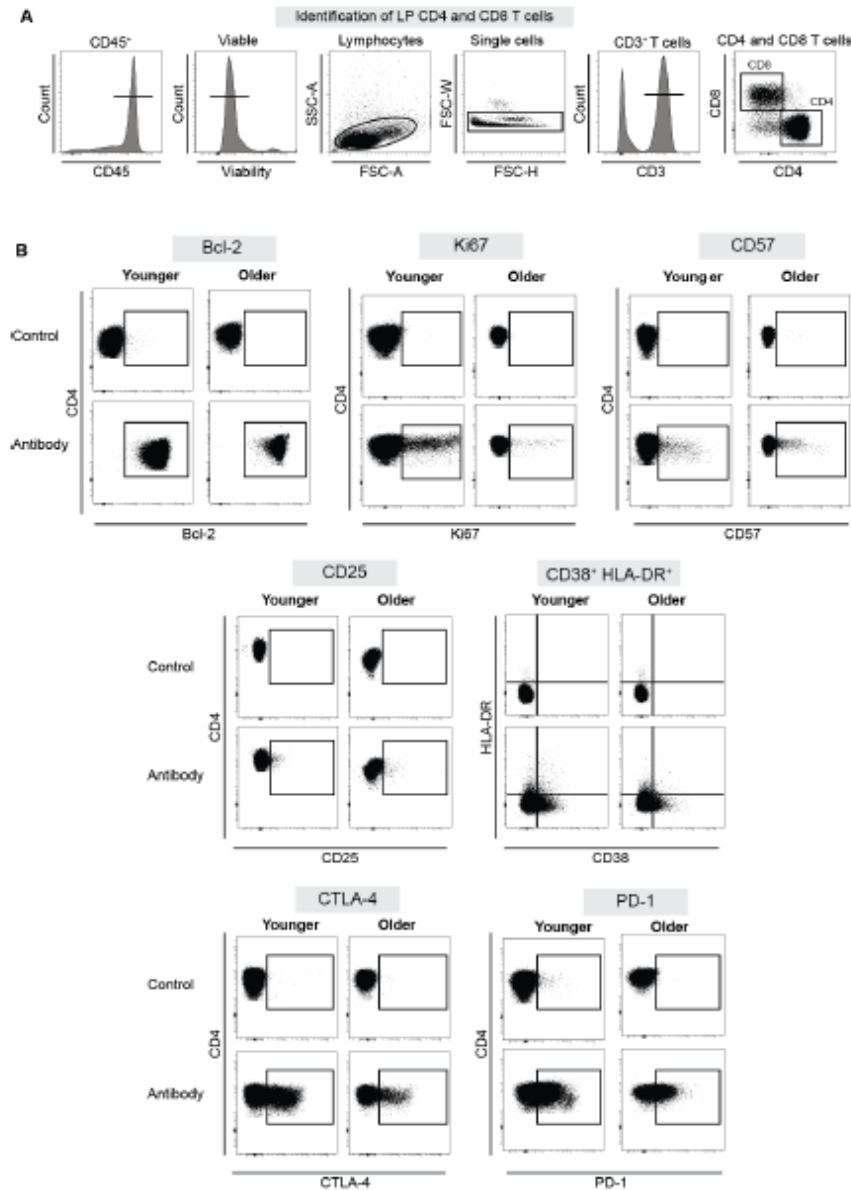

Supplement: Supplementary file 1 — Additional file 1: Figure S1. Multi-color flow cytometry profiles to enumerate frequencies and phenotypic profiles of human colon LP CD4 T cells. [file 12979_2021_217_MOESM1_ESM.pdf]
