# Supplementary material for: Reduced immune-regulatory molecule expression on human colonic memory CD4 T cells in older adults
Source: Immun Ageing. 2021 Feb 13;18:6. doi: 10.1186/s12979-021-00217-0 (PMC7881462; doi:10.1186/s12979-021-00217-0)
Supplement: Supplementary file 2 — Additional file 2: Figure S2. LP and PB memory CD4 T cell profiles. [file 12979_2021_217_MOESM2_ESM.pdf]

Additional File 2.

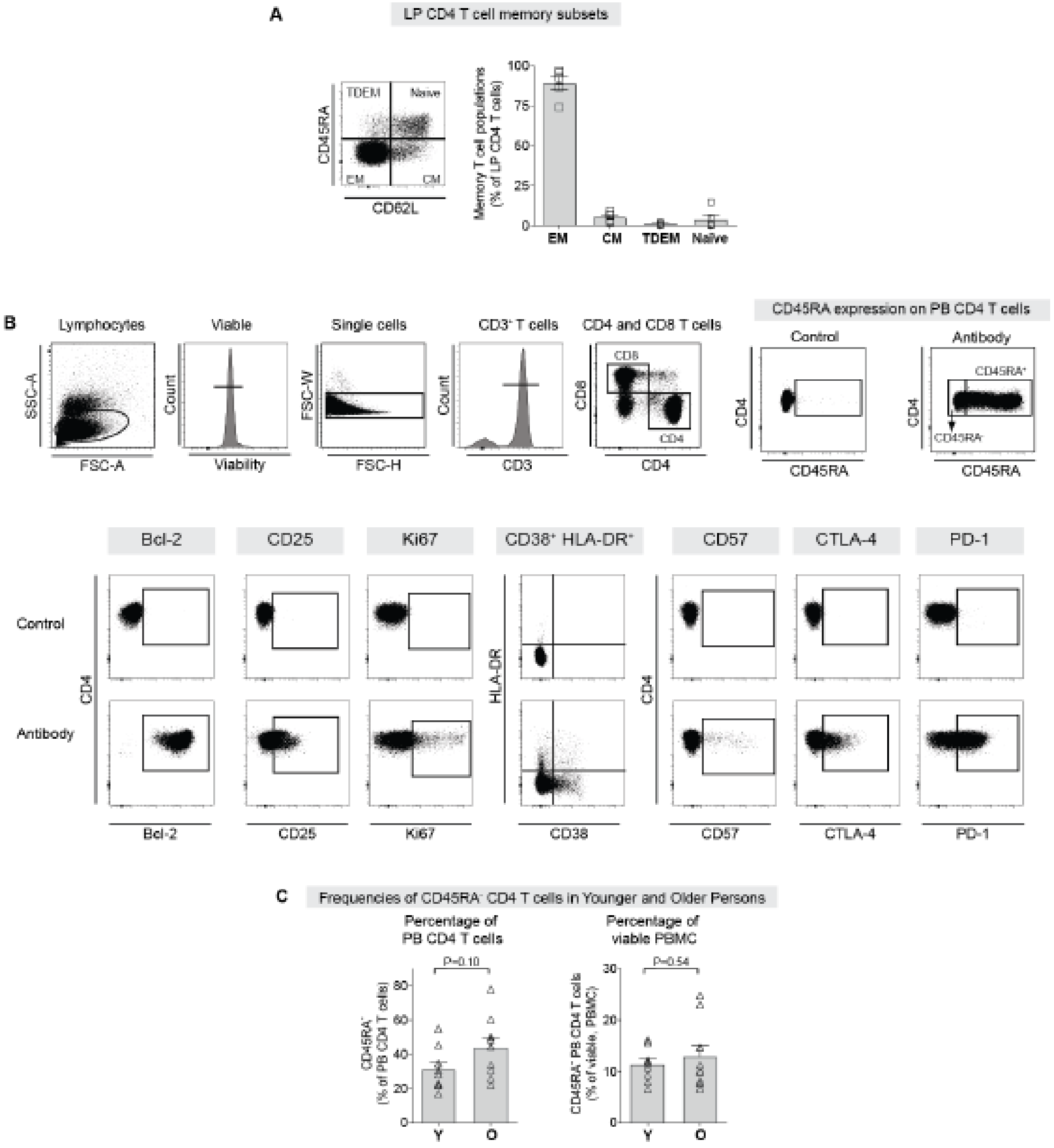

Figure S2. LP and PB memory CD4 T cells. a Multi-color flow cytometry was used to enumerate frequencies of LP CD4 memory T cells defined as Effector Memory (EM; $CD45RA^{-}CD62L^{-}$), Central Memory (CM, $CD45RA^{-}CD62L^{+}$), Terminally Differentiated Effector Memory (TDEM; $CD45RA^{+}CD62L^{-}$) and Naïve ($CD45RA^{+}CD62L^{+}$) subsets. Representative flow plot (left panel) and the distribution of LP memory CD4 T cell

subsets in LPMC (N=5) with bar graphs representing mean ± SEM and individual samples shown as open squares. Isotype controls were used to establish CD45RA vs CD62L gating. **b** Representative gating strategy to identify PB CD45RA$^-$ CD4 T cells within viable, single CD3$^+$ lymphocytes and expression of various markers by PB CD45RA$^-$ CD4 T cells from a younger person. FSC: forward scatter, SSC: side scatter; A: Area, H: Height; W: Width. An isotype control was used to establish CD45RA$^+$ staining and analyses were performed on CD45RA$^-$ cells (highlighted by the arrow). For phenotypic analyses, gates established were on Control staining (upper panel: isotype control for Bcl-2, Ki67, CD38, CTLA-4, HLA-DR and PD-1 and Fluorescence minus one (FMO) for CD25 and CD57. **c** Comparisons of frequencies of PB CD45RA$^-$CD4$^+$CD8$^-$ T cells in younger (Y; N=9) and older (O; N=9) persons determined as a percentage of CD4 T cells (left panel) and as the percentage of CD45RA$^-$ CD4 T cells within viable PBMC (right panel). A large FSC versus SSC gate was used to establish total PMBC and viable cells within this gate determined (not shown). Statistical analysis: unpaired t-test.
