# Supplementary material for: Reduced immune-regulatory molecule expression on human colonic memory CD4 T cells in older adults
Source: Immun Ageing. 2021 Feb 13;18:6. doi: 10.1186/s12979-021-00217-0 (PMC7881462; doi:10.1186/s12979-021-00217-0)
Supplement: Supplementary file 3 — Additional file 3: Figure S3. Multi-color flow cytometry profiles to enumerate frequencies and phenotypic profiles of human colon LP CD8 T cells. [file 12979_2021_217_MOESM3_ESM.pdf]

Additional File 3.

A

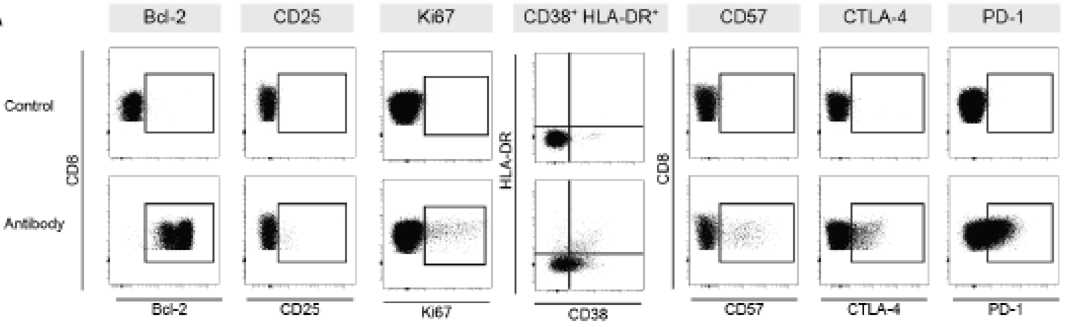

B

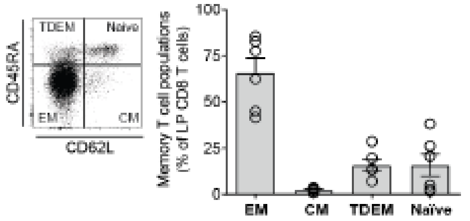

C

LP CD8 and LP CD4 T cell memory subsets in younger persons.

	LP CD8 T cells (N=6)	LP CD4 T cells (N=5)	P value
**EM**	65.9 ± 7.9%	89.1 ± 4.2%	**P=0.007**
**CM**	2.4 ± 0.5%	5.5 ± 1.3%	**P=0.04**
**TDEM**	15.9± 3.0%	1.2 ± 0.4%	**P=0.004**
**Naïve**	15.8 ± 6.1%	4.2 ± 2.8%	P=0.10

Values are shown as the mean±SEM of the percentage of effector memory (EM; $CD62L^-CD45RA^-$), central memory (CM; $CD62L^+CD45RA^-$), terminally differentiated effector memory (TDEM; $CD62L^-CD45RA^+$) and naïve ($CD62L^+CD45RA^+$) cells in LP CD8 or LP CD4 T cells. Statistical analyses: paired t test. Bold values indicate statistically significant difference between LP CD8 and LP CD4 T cells.

Figure S3. Multi-color flow cytometry was used to enumerate frequencies and phenotypic profiles of human colonic LP CD8 T cells. **a** Representative flow plots for expression of various markers by LP CD8 T cells from a younger person with gates established on Control staining (upper panel: isotype control for Bcl-2, Ki67, CD38 and CTLA-4, Fluorescence minus one (FMO) for CD25 or FM4 for HLA-DR, CD57 and PD-1). **b** Frequencies of LP CD8 memory T cells defined as Effector Memory (EM; $CD45RA^-CD62L^-$), Central Memory (CM, $CD45RA^-CD62L^+$), Terminally Differentiated Effector Memory (TDEM; $CD45RA^+CD62L^-$) and Naïve ($CD45RA^+CD62L^+$) subsets. Representative flow plot (left panel) and the distribution of LP memory CD8 T cell subsets (N=6) with bar graphs representing mean ± SEM and individual samples shown as open circles. **c** Comparisons of LP CD8 (N=6) and LP CD4 (N=5) Memory and Naïve T cell subsets in younger persons. Statistical analysis: paired t-test.
