# Supplementary material for: Reduced immune-regulatory molecule expression on human colonic memory CD4 T cells in older adults
Source: Immun Ageing. 2021 Feb 13;18:6. doi: 10.1186/s12979-021-00217-0 (PMC7881462; doi:10.1186/s12979-021-00217-0)
Supplement: Supplementary file 4 — Additional file 4: Table S1. Frequencies and expression of survival, activation and immune-regulatory markers by LP CD8 T cells or LP CD4 T cells in younger persons. [file 12979_2021_217_MOESM4_ESM.pdf]

Additional File 4.

Table S1. Frequencies and expression of survival, activation and immune-regulatory markers by LP CD8 T cells or LP CD4 T cells in younger persons.

**Phenotypic Marker**	**LP CD8 T cells (N=9)**	**LP CD4 T cells (N=9)**	**P Value**
Frequency of viable, $CD45^+$ LPMC (%)	14.0 ± 0.29	41.1 ± 3.0	**0.0004**
Absolute number (per gram of mucosa)	624340 ± 164682	2084054 ± 601302	**0.02**
Percentage of $CD3^+$ T cells (%)	22.3 ± 3.9	70.1 ± 4.4	**0.0004**
Bcl-2 expression level (GMFI)*	7316 ± 925	9147 ± 727	**<0.05**
$CD38^+HLA\text{-}DR^+$ (%)$^\#$	5.8 ± 1.3†	1.6 ± 0.4†	**0.008**
CD25 (%)	1.8 ± 0.4	5.1 ± 1.3	**0.01**
Ki67 (%)	7.0 ± 1.7	5.5 ± 1.2	0.46
CD57 (%)	11.5 ± 3.7†	3.7 ± 0.7†	**0.04**
CTLA-4 (%)	15.2 ± 3.8	44.8 ± 6.6†	**<0.0001**
CTLA-4 expression level (GMFI)	59 ± 9	237 ± 16†	**<0.0001**
PD-1 (%)	27.9 ± 4.0†	56.3 ± 3.4†	**<0.0001**
PD-1 expression level (GMFI)	615 ± 105†	1173 ± 161†	**0.0003**

*Geometric mean fluorescence intensity (GMFI); $^\#$percentage of LP CD8 or LP CD4 T cells. Net values (control staining values removed) are shown as mean ± SEM. Statistical analysis: paired t-test. Bold values indicate statistically significant differences between LP CD8 T cells and LP CD4 T cells. †N=8.
