# Supplementary material for: Reduced immune-regulatory molecule expression on human colonic memory CD4 T cells in older adults
Source: Immun Ageing. 2021 Feb 13;18:6. doi: 10.1186/s12979-021-00217-0 (PMC7881462; doi:10.1186/s12979-021-00217-0)
Supplement: Supplementary file 5 — Additional file 5: Table S2. Patient details for procured colonic tissue samples. [file 12979_2021_217_MOESM5_ESM.pdf]

Additional File 5.

Table S2. Patient details for procured colonic tissue samples.

Sample ID	Age (yrs)	Sex	Reason for elective surgery
Samples used for phenotypic studies			
Younger			
G143	28	M	Diverticulitis (not active)
G217	41	M	Rectal cancer
G268	35	M	Rectal cancer
G329	41	F	Colon cancer
G344	40	F	Neuroendocrine tumor
G350	39	F	Gastrointestinal Stromal Tumor
G366	27	M	Colon cancer
G415	41	M	Familial Adenomatous Polyposis
G477	44	F	Rectal cancer
Older			
G73	80	M	Rectal cancer
G246	88	F	Colon cancer
G298	75	F	Sarcoma
G337	85	M	Vovullus
G345	85	M	Colon cancer
G375	72	F	Gastric cancer
G401	80	M	Appendix Cancer
G409	80	M	No details recorded
G505	79	M	Colon cancer
Samples used for measurement of memory CD4 and CD8 T cell populations.			
G71	32	F	Rectal cancer
G99	43	F	Temporary diverting loop
G133	41	F	Diverticular disease (not active)
G135	44	M	Diverticulitis (not active)
G142	36	M	Diverticulitis (not active)
G160	43	F	Radiation induced stricture of the rectum

M: Male; F: Female.
