# Supplementary material for: Reduced immune-regulatory molecule expression on human colonic memory CD4 T cells in older adults
Source: Immun Ageing. 2021 Feb 13;18:6. doi: 10.1186/s12979-021-00217-0 (PMC7881462; doi:10.1186/s12979-021-00217-0)
Supplement: Supplementary file 6 — Additional file 6: Table S3. Antibodies and dyes used for multi-color flow cytometry. [file 12979_2021_217_MOESM6_ESM.pdf]

Additional File 6.

Table S3. Antibodies and dyes used for multi-color flow cytometry.

Antibody	Fluorochrome	Clone	Company
CD4 and CD8 T cell identification for phenotypic studies			
Viability	Zombie Aqua Dye		BioLegend[1]
CD45	BV785	H130	BioLegend
CD3	Vfluor[450]	OKT3	Tonbo Biosciences[2]
CD4	BUV395	SK3	BD Biosciences[3]
CD8	PerCpCy5.5	RPA-T8	Tonbo Biosciences
PB Memory T cells			
CD45RA	BV785	HI100	Biolegend
Mouse IgG2b	BV785	MPC-11	Biolegend
Cell Survival			
Bcl-2	PE	100	BioLegend
Mouse IgG1	PE	MOPC-21	BioLegend
Homeostatic proliferation			
Ki67	PE-Cy7	B56	BD Biosciences
Mouse IgG1	PE-Cy7	MOPC-21	BD Biosciences
Cell senescence			
CD57	APC	HCD57	BioLegend
Cell activation			
CD38	AF700	HIT2	Invitrogen[4]
Mouse IgG1	AF700	P3.6.2.8.1	Invitrogen
HLA-DR	APC-Cy7	L243	BioLegend
Mouse IgG1	APC-Cy7	MOPC-173	Biolegend
CD25	APC-Cy7	M-A251	BD Biosciences
Negative regulation			
CTLA-4	APC	BNI3	Tonbo Biosciences
Mouse IgG2a	APC	C1.18.4	Tonbo Biosciences
PD-1	PE-Cy7	EH12.2H7	BioLegend
Mouse IgG1	PE-Cy7	MOPC-21	Biolegend
LAG-3	FITC	11C3C65	BioLegend
Mouse IgG1	FITC	MOPC-21	BioLegend
Tim-3	PE	F38-2E2	BioLegend
Mouse IgG1	PE	MOPC-21	BioLegend
Colon Memory CD4 and CD8 T cell subsets			
Viability	Zombie Aqua Dye		BioLegend
CD45	PerCp-Cy5.5	2D1	Invitrogen
CD3	PE-Texas Red (ECD)	UCHT1	Beckman Coulter[5]
CD4	AF700	RPA-T4	BD Biosciences
CD8	efluor[450]	RPA-T8	Invitrogen
CD45RA	FITC	HI100	Biolegend
PE CD62L	PE	DREG-56	Biolegend

[1]BioLegend, San Diego, CA. [2]Tonbo Biosciences, San Diego, CA. [3]BD Biosciences, San Jose, CA. [4]Invitrogen (eBioscience) Thermo-Fisher Scientific, Waltham, MA. [5]Beckman Coulter, Brea, CA.
